# Two new 2-alkylquinolones, inhibitory to the fish skin ulcer pathogen *Tenacibaculum maritimum*, produced by a rhizobacterium of the genus *Burkholderia* sp.

**DOI:** 10.3762/bjoc.14.122

**Published:** 2018-06-14

**Authors:** Dandan Li, Naoya Oku, Atsumi Hasada, Masafumi Shimizu, Yasuhiro Igarashi

**Affiliations:** 1 Biotechnology Research Center and Department of Biotechnology, Toyama Prefectural University, 5180 Kurokawa, Imizu, Toyama 939-0398, Japan; 2 Laboratory of Plant Pathology, Faculty of Applied Biological Sciences, Gifu University, 1-1 Yanagido, Gifu 501-1193, Japan

**Keywords:** antimicrobial, *Burkholderia*, quinolone, skin ulcer, *Tenacibaculum maritimum*

## Abstract

Exploration of rhizobacteria of the genus *Burkholderia* as an under-tapped resource of bioactive molecules resulted in the isolation of two new antimicrobial 2-alkyl-4-quinolones. (*E*)-2-(Hept-2-en-1-yl)quinolin-4(1*H*)-one (**1**) and (*E*)-2-(non-2-en-1-yl)quinolin-4(1*H*)-one (**3**) were isolated from the culture broth of strain MBAF1239 together with four known alkylquinolones (**2** and **4**–**6**), pyrrolnitrin (**7**), and BN-227 (**8**). The structures of **1** and **3** were unambiguously characterized using NMR spectroscopy and mass spectrometry. Compounds **1**–**8** inhibited the growth of the marine bacterium *Tenacibaculum maritimum*, an etiological agent of skin ulcers in marine fish, offering new opportunities to develop antibacterial drugs for fish farming.

## Findings

Bacteria of the genus *Burkholderia* within the family *Burkholderiaceae* [1], along with their neighboring genera, *Paraburkholderia*, *Caballeronia* [2], and *Robbsia* [3] constitute a distinct group of bacteria within the class *Betaproteobacteria*. These bacteria are obligatory aerobic, mostly motile, non-spore-forming Gram-negative rods of strictly terrestrial origin. Although isolation of *Burkholderia* from marine sediments has been reported, these bacteria may not originate in the marine environment, as enrichment in low salinity media is a prerequisite for isolation [4–6]. Most of them live in close association with animals or plants as pathogens or symbionts and exhibit a variety of catabolic and metabolic activities [1,7].

One hundred ten secondary metabolites have been reported from *Burkholderia* (data retrieved from the Dictionary of Natural Products, as of March 20, 2018). However, it is likely that *Burkholderia* produce many more secondary metabolites than reported, as this group was previously classified into the genus *Pseudomonas* [8]. In fact, the high capacity of *Burkholderia* in secondary metabolism is demonstrated by the presence of unique functionalities, such as monocyclic 3-pyrazolone [9], α-aminoacrylonitrile, and thioimidazolinone [10], all of which are not preceded in metabolites from other taxa.

The large genome size of *Burkholderia* also suggests a high capacity for secondary metabolism. According to the NCBI genome database (https://www.ncbi.nlm.nih.gov/genome/browse#!/prokaryotes/), the genome sizes of *B. cepacia* ATCC25416, *Paraburkholderia terrae* DSM 17804, and *Caballeronia glathei* DSM50014 are 8.61, 10.1, and 8.64 Mbp, respectively, which are comparable to 9.05 Mbp for *Streptomyces coelicolor* A3(2) and 9.14 Mbp for *Myxococcus xanthus* DK 1622, both known as representatives of prolific antibiotic producers (as of Jan. 20, 2018).

**Figure 1:**
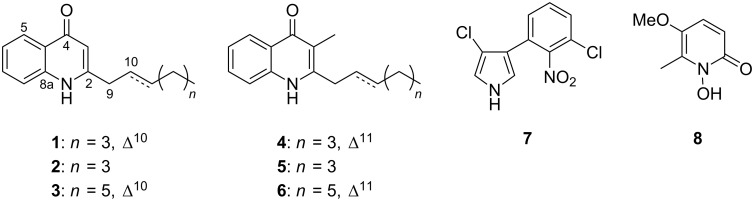
Structures of compounds **1**–**8**.

As part of our program to further explore this unique pharmacological resource, rhizobacteria of the genus *Burkholderia* were collected and tested for the production of antimicrobial metabolites against a panel of plant and animal pathogens consisting of 4 bacteria, 1 yeast, and 4 fungi. The result of this screening prompted the detailed chemical study of a strain coded as MBAF1239, which resulted in the isolation of eight antibacterial metabolites, including the two new 2-alkylquinolones **1** and **3** (Figure 1).

Strain MBAF1239 was seed-cultured in V22 medium and subsequently transferred into IMM-HS medium, which was designed for metabolite production based on the composition of IMM [11] and HS media [12]. The fermented culture was extracted with 1-BuOH and the extract was fractionated by solvent-partitioning to yield *n*-hexane-, 90% aqueous MeOH-, and 60% aqueous MeOH-soluble fractions. Antimicrobial testings against *Rhizopus oryzae* (the fungal pathogen of rice seedling blight), *Trichophyton rubrum* (dermatophytosis pathogen), and *Tenacibaculum maritimum* (the causative organism for skin ulcers in marine fish) revealed that the second fraction was the most active. The second fraction was then further fractionated by ODS flash chromatography and purified by HPLC to yield the eight metabolites **1**–**8**.

**Figure 2:**
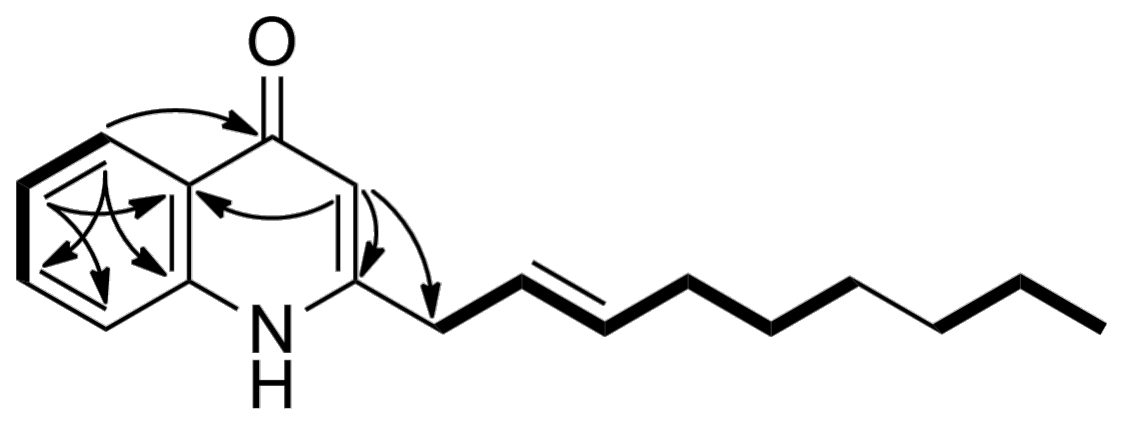
Key COSY (bold line) and HMBC (arrow) correlations for **3**.

The molecular formula of **3** was established as C_18_H_23_NO based on a HRESITOFMS measurement (*m/z* 270.1855 [M + H]^+^, Δ +0.3 mmu). Analysis of ^1^H, ^13^C, and HSQC NMR spectra in CDCl_3_ (Supporting Information File 1, Figures S6, S7, and S9) revealed four aromatic (δ_H_/δ_C_ 8.34/126.3, 7.57/131.9, 7.32/123.6, and 7.26/116.8) and three olefinic (δ_H_/δ_C_ 6.18/109.5, 5.79/137.7, and 5.54/123.0) methines, six aliphatic methylenes (δ_H_/δ_C_ 3.37/37.4, 2.12/32.5, 1.43/29.1, 1.33/28.9, 1.31/22.6, and 1.30/31.7), and a methyl (δ_H_/δ_C_ 0.89/14.1) group, leaving one carbonyl (δ_C_ 179.0) and three aromatic resonances (δ_C_ 149.9, 139.4, and 125.3) as quaternary carbons (Table 1). Because these structural elements accounted for six out of eight degrees of unsaturation, the remaining two degrees correspond to two rings, which constitutes a fused bicyclic structure as suggested by the number of available aromatic carbons (eleven). A 4-quinolone substructure was indicated by a peak-splitting at the 340–320 nm region in the UV spectrum (328 and 322 nm) [13]. Indeed, ^1^H NMR resonances at the down field region was superimposable on those of known 2-heptyl-4(1*H*)-quinolone (**2**, Supporting Information File 1, Figure S11). The COSY and HMBC correlations also supported this assignment (Figure 2; Supporting Information File 1, Figures S8 and S10). An extension of a 2-nonenyl group (C9–C17) at C2 was supported by an HMBC correlation from H3 to C9 (Figure 2).

**Table 1:** NMR data for (*E*)-2-(non-2-en-1-yl)quinolin-4(1*H*)-one (**3**) in CDCl_3_ (δ in ppm).

pos.	δ_C_	δ_H_, mult. (*J* in Hz), integr.	COSY	HMBC (^1^H to ^13^C)

1		8.07, br, 1H		
2	149.9			
3	109.5	6.18, s, 1H		2, 4a, 9
4	179.0			
4a	125.3			
5	126.3	8.34, d (7.9), 1H	6	4, 7, 8a
6	123.6	7.32, t, 7.5, 1H	5, 7	4a, 8
7	131.9	7.57, brs, 1H	6	
8	116.8	7.26, ovl^a^		
8a	139.4			
9	37.4	3.37, brs, 2H	10	
10	123.0	5.54, m, 1H	9, 11	
11	137.7	5.79, m, 1H	10,12	
12	32.5	2.12 ddd (7.2, 6.5, 6.3), 2H	11, 13	10, 11, 13, 14
13	29.1	1.43, m, 2H	12, 14	
14	28.9	1.33, m, 2H		
15	31.7	1.30, m, 2H		
16	22.6	1.31, m, 2H	17	
17	14.1	0.89, t (6.5), 3H	16	15, 16

^a^Signal overlapped by a residual solvent peak.

**Figure 3:**
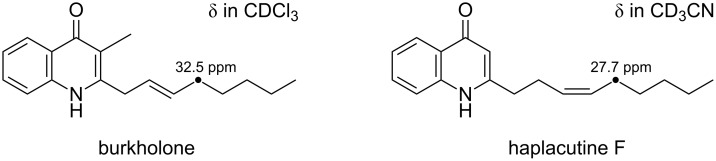
Referential ^13^C chemical shifts of an allylic carbon in burkholone [14] and haplacutine F [15].

The obtuse ^1^H NMR signal shapes, presumably due to limited solubility of **3** in CDCl_3_, hampered unequivocal determination of the C10-geometry based on a coupling constant between the olefinic protons H10 and H11. Instead, a lack of NOESY correlation between these protons was indicative of an *E*-geometry (Supporting Information File 1, Figure S12). This was finally supported by a chemical shift value for the C12 allylic carbon at δ 32.5, which is closer to that of an *E*-isomer, burkholone (δ 32.5) [14], than that of an *Z*-isomer, haplacutine F (δ 27.7) [15] (Figure 3). Thus, the structure of **3** was concluded to be (*E*)-2-(non-2-en-1-yl)quinolin-4(1*H*)-one.

The molecular ions of **1** were observed at *m*/*z* 242 and *m*/*z* 240 in the positive and negative modes, respectively, revealing a 28 Da smaller molecular weight for **1** relative to **3**. The ^1^H NMR spectra of both compounds (Supporting Information File 1, Figures S6 and S1) are similar except for the integral of the methylene resonances between 1.48 and 1.23 ppm. While the resonance amounted to 8H-equivalent in **3**, the resonance in **1** was equivalent to 4H, suggesting that **1** is a congener of **3** with a two-methylene shorter appendage. This was later confirmed by the interpretation of COSY, HSQC, and HMBC spectra (Supporting Information File 1, Figures S3–S5), allowing the full assignment of ^1^H and ^13^C NMR resonances (see Experimental). Thus, **1** was determined to be (*E*)-2-(hept-2-en-1-yl)quinolin-4(1*H*)-one.

The remaining metabolites were identified as known compounds based on NMR and MS data (data not shown). Compound **2** was identified as Pyo Ib or 2-heptylquinolin-4(1*H*)-one [16], **4** was identified as (*E*)-2-(hept-2-en-1-yl)-3-methylquinolin-4(1*H*)-one [13], **5** was identified as PSC-C or 2-heptyl-3-methylquinolin-4(1*H*)-one [17], **6** was identified as PSC-D or (*E*)-3-methyl-2-(non-2-en-1-yl)quinolin-4(1*H*)-one) [17], **7** was identified as pyrrolnitrin [18], and **8** was identified as BN-227 [19] (Figure 1).

4-Quinolone is a common core in synthetic antibactericides [20] and in bioactive metabolites produced by *Rutaceae* plants [21–25], Gram-positive [26–27] and Gram-negative bacteria [28–30], and a marine sponge [31]. Among a variety of substituted 4-quinolones, 2-alkyl-4-quinolones are the most common core in antibiotics [32], which were originally discovered as anti-anthrax metabolites produced by *Pseudomonas aeruginosa*. A series of chemoecological studies of *P. aeruginosa* has uncovered multifunctional roles of this quinolone class as antibacterial, antifungal, iron-chelating, and autoinducer agents to assist the survival of the producing organisms [32]. Additionally, drug discovery attempts have revealed 5-lipoxygenase inhibitory activity [33], plant-growth promoting activity [32,34], and IGF-dependent cell-specific cytotoxicity [14].

A recent metabolomic analysis using LC–MS verified the presence of more than 50 2-alkyl-4-quinolones [35], most of which remain chemically and biologically uncharacterized. Compounds **1** and **3** are among these uncharacterized analogs, with their (presumable) detection by mass spectrometry reported twice [29,36] and three times [36–38], respectively. In this study, we have isolated both compounds for the first time, which enabled rigorous structure characterization, including the position and geometry of unsaturation in the side chains, as well as evaluation of their bioactivity (see below).

Compounds **1**–**8** at 10 μg/disc inhibited the growth of a bacterium of the phylum *Bacteroidetes*, *T. maritimum* (Table 2). Overall, alkylquinolones **1**–**6** were more potent than **7** and **8**. Among **1**–**6**, the 2-heptenyl-3-methyl congener **4** was the most active. Compounds **3**–**6** also inhibited the growth of the fungi *R. oryzae* and *T. rubrum*, while **1** and **2** did not. This may to some extent attributable to the global lipophilicity of molecules, as **1**and **2** are among the fastest eluting congeners during the reversed-phase separation. Because *T. maritimum* is one of the major etiologies for fatal skin ulcers in marine fish [39], **1**–**8** could offer novel scaffolds to develop new therapeutic modalities for this economically devastating epizootic.

**Table 2:** Antimicrobial activity of **1**–**8** evaluated at 10 μg on Ø 6 mm-paper disc.

	*Tenacibaculum maritimum*	*Trichophyton rubrum*	*Rhizopus oryzae*

**1**	33^a^	0	0
**2**	24	0	0
**3**	25	30	10
**4**	55	34	10
**5**	22	20	30
**6**	25	30	15
**7**	20	–^b^	–
**8**	7	–	–

^a^Size of inhibitory zone in mm. ^b^Not tested.

## Experimental

### General experimental procedures

UV and IR spectra were recorded on a Hitachi U-3210 and a Perkin Elmer Spectrum 100 spectrophotometer, respectively. ^1^H and ^13^C NMR spectra were obtained on a Bruker AVANCE 500 spectrometer referencing solvent peaks at δ_H_/δ_C_ 7.26/77.0 ppm for CDCl_3_ and δ_H_/δ_C_ 3.30/49.0 ppm for CD_3_OD. ESITOFMS spectra were collected on a Bruker micrOTOF focus mass spectrometer.

### Collection of *Burkholderia* strains and broth screening

*Burkholderia* strains were collected by serial dilution plating on Pseudomonas agar supplemented with C-F-C (Oxoid, Basingstoke, England) from rhizosphere soils of Welsh onion and cucumber (*Cucumis sativus*), grown in an experimental farm at Mie University (Kurimamachiya-cho, Tsu, Mie) in 2010. The bacterial collection was cultured in 4 different media to give 152 extracts, which were screened against *Edwardsiella ictaluri* NBRC105724^T^ (pathogen of enteric septicemia of freshwater fish), *T. maritimum* NBRC16015, *Trichophyton rubrum* NBRC5467, *Candida albicans* NBRC0197 (human opportunistic pathogen), *R. oryzae* NBRC4705, *Glomerella cingulata* NBRC5907 (pathogen of anthracnose), *Ralstonia solanacearum* SUPP1541 (pathogen of bacterial wilt of *Solanaceous* plants), *Rhizobium radiobacter* NBRC14554 (pathogen of crown gall), *Athelia rolfsii* NBRC30071 (pathogen of southern blight). All but two strains, or 139 extracts out of 152, exhibited activity against at least one pathogen, demonstrating an impressively high incidence of antagonistic strains in this genus. One of the prominent hit extracts was prepared from a culture of strain MBAF1239, isolated from a rhizosphere of Welsh onion (*Allium fistulosum*). A 16S rDNA sequence analysis identified MBAF1239 as *Burkholderia* sp. within the *B. cepacia* complex (DDBJ accession number LC194190).

### Fermentation, extraction, and isolation

*Burkholderia* sp. MBAF1239 was seed-cultured in 500 mL K-1 flasks each containing 100 mL of medium V-22 (soluble starch 1%, glucose 0.5%, NZ-case 0.3%, yeast extract 0.2%, tryptone 0.5%, K_2_HPO_4_ 0.1%, MgSO_4_·7H_2_O 0.05%, and CaCO_3_ 0.3%, pH 7.0) by rotary shaking at 200 rpm at 30 °C for two days. A three-mL aliquot of the resulting culture was inoculated into 100 mL of the IMM-HS production medium (glucose 1%, K_2_HPO_4_ 0.36%, KH_2_PO_4_ 0.41%, MgSO_4_·7H_2_O 0.02%, CaCl_2_·2H_2_O 0.01%, FeSO_4_·7H_2_O 0.002%, NH_4_Cl 0.1%, biotin 0.0001%, and L-histidine 0.4%), and shaken at 200 rpm at 30 °C for 4 days.

For the extraction of secondary metabolites, 100 mL of 1-butanol was added to each flask, and they were allowed to shake for 1 h. The resulting suspension was centrifuged at 6000 rpm for 10 min to separate organic and aqueous layers, the former of which was concentrated in vacuo to give a 5.35 g extract from a 2 L culture. The crude extract was successively partitioned between 60% MeOH (250 mL) and CH_2_Cl_2_ (250 mL × 3) and the latter between 90% aqueous MeOH (150 mL) and *n*-hexane (150 mL × 3). The aqueous MeOH layer, which concentrated the antibacterial activity against *Tenacibaculum maritimum*, was subjected to ODS flash chromatography (Ø 3 × 7 cm) eluted with a stepwise gradient of 25, 40, 55, and 85% (v/v) MeCN in 50 mM NaClO_4_. The most active third fraction (37.4 mg) was purified by reversed-phase HPLC on a Cosmosil AR-II column (Ø 1 × 25 cm) with a linear gradient elution program [eluents: MeOH (A), 1:1 CH_3_CN/H_2_O (B); 0–5 min 100% B, 5–45 min 100% B to 0% B, 45–65 min 0% B; flow 3 mL min^−1^; UV detection at 210 nm] to afford two new 2-alkylquinolones **1** (0.5 mg) and **3** (0.7 mg), together with four known 2-alkylquinolones, Pyo Ib or 2-heptylquinolin-4(1*H*)-one (**2**, 2.3 mg), (*E*)-2-(hept-2-en-1-yl)-3-methylquinolin-4(1*H*)-one (**4**, 2.5 mg), PSC-C or 2-heptyl-3-methylquinolin-4(1*H*)-one (**5**, 1.0 mg), PSC-D or (*E*)-3-methyl-2-(non-2-en-1-yl)quinolin-4(1*H*)-one (**6**, 0.8 mg), pyrrolnitrin (**7**, 0.5 mg), and BN-227 (**8**, 0.8 mg).

**(*E*)-2-(Hept-2-en-1-yl)quinolin-4(1*H*)-one (1):** UV (MeCN) λ_max_, nm (ε): 328 (29900), 322 (27600), 316 (29800), 292 (10700), 288 (10900), 260 (6600), 240 (51200); IR ν _max_ (ATR) cm^−1^: 2927, 2873, 1636, 1595, 1553, 1505, 1473, 1445, 1355, 1322, 1275, 1104, 1028, 969, 841, 762, 676; ^1^H NMR (CD_3_OD) δ_H_ 8.20 (d, *J* = 8.1 Hz, 1H, H5), 7.68 (brs, 1H, H7), 7.57 (brs, 1H, H8), 7.39 (brt, *J* = 6.9 Hz 1H, H6), 6.21 (s, 1H, H3), 5.71 (m, 1H, H11), 5.61 (brd, *J* = 13.9 Hz, 1H, H10), 3.42 (brs, 2H, H9), 2.09 (dt, *J* = 6.3 and 6.2 Hz, 2H, H12), 1.38 (m, 2H, H13), 1.34 (m, 2H, H14), 0.90 (t, *J* = 6.9 Hz, 3H, H15); ^13^C NMR (CD_3_OD) δ_C_ 180.8 (C4), 155.7 (C2), 141.6 (C8a), 136.5 (C11), 133.5 (C7), 126.0 (C5), 125.5 (C4a), 125.3 (C10), 125.1 (C6), 119.1 (C8), 108.9 (C3), 37.8 (C9), 33.2 (C12), 32.6 (C13), 23.2 (C14), 14.2 (C15); ESIMS–TOF (*m/z*): [M + H]^+^ calcd for C_16_H_19_NO, 242.1539; found, 242.1539.

**(*E*)-2-(Non-2-en-1-yl)quinolin-4(1*H*)-one (3):** UV (MeCN) λ_max_, nm (ε): 328 (18600), 322 (16800), 316 (18200), 292 (8000), 288 (8400), 260 (5300), 240 (23,200); IR ν _max_ (ATR) cm^−1^: 2923, 2853, 1730, 1635, 1593, 1554, 1500, 1471, 1443, 1354, 1320, 1247, 1137, 1028, 965, 836, 759, 672; HRESIMS–TOF (*m/z*): [M + H]^+^ calcd for C_18_H_24_NO, 270.18524; found, 270.1855.

### Evaluation of antimicrobial activity

The antibacterial and antifungal activity of **1**–**8** was evaluated by a paper-disc agar diffusion method described in our previous study [40]. Flexibacter maritimus medium (0.5% peptone and 0.05% yeast extract in sea water) solidified with 10% agar was used to test against *T. maritimum*.

## Supporting Information

File 1 ^1^H and ^13^C NMR, COSY, HSQC, and HMBC spectra for compounds **1** and **3**.
