# Supplementary material for: Two new 2-alkylquinolones, inhibitory to the fish skin ulcer pathogen Tenacibaculum maritimum, produced by a rhizobacterium of the genus Burkholderia sp
Source: Beilstein J Org Chem. 2018 Jun 14;14:1446–51. doi: 10.3762/bjoc.14.122 (PMC6009182; doi:10.3762/bjoc.14.122)
Supplement: File 1 — 1H and 13C NMR, COSY, HSQC, and HMBC spectra for compounds 1 and 3. [file Beilstein_J_Org_Chem-14-1446-s001.pdf]

Supporting Information

for

Two new 2-alkylquinolones, inhibitory to the fish skin ulcer pathogen *Tenacibaculum maritimum*, produced by a rhizobacterium of the genus *Burkholderia* sp.

Dandan Li[1], Naoya Oku[1], Atsumi Hasada[1], Masafumi Shimizu[2], and Yasuhiro Igarashi*[1]

Address: [1]Biotechnology Research Center and Department of Biotechnology, Toyama Prefectural University, 5180 Kurokawa, Imizu, Toyama 939-0398, Japan and [2]Laboratory of Plant Pathology, Faculty of Applied Biological Sciences, Gifu University, 1-1 Yanagido, Gifu 501-1193, Japan

Email: Yasuhiro Igarashi* - yas@pu-toyama.ac.jp

* Corresponding author

^{1}H and ^{13}C NMR, COSY, HSQC, and HMBC spectra for compounds 1 and 3

(*E*)-2-(hept-2-en-1-yl)quinolin-4(1*H*)-one (**1**)

^{1}H NMR spectrum (CH_3OD, 500 MHz) S2

^{13}C NMR spectrum (CH_3OD, 125 MHz) S3

COSY spectrum (CH_3OD, 500 MHz) S4

HSQC spectrum (CH_3OD, 500 MHz) S5

HMBC spectrum (CH_3OD, 500 MHz) S6

(*E*)-2-(non-2-en-1-yl)quinolin-4(1*H*)-one (**3**)

^{1}H NMR spectrum ($CDCl_3$, 500 MHz) S7

^{13}C NMR spectrum ($CDCl_3$, 125 MHz) S8

COSY spectrum ($CDCl_3$, 500 MHz) S9

HSQC spectrum ($CDCl_3$, 500 MHz) S10

HMBC spectrum ($CDCl_3$, 500 MHz) S11

^{1}H NMR spectral comparison with 2-heptylquinolin-4(1*H*)-one (**2**) S12

NOESY spectrum ($CDCl_3$, 500 MHz) S13

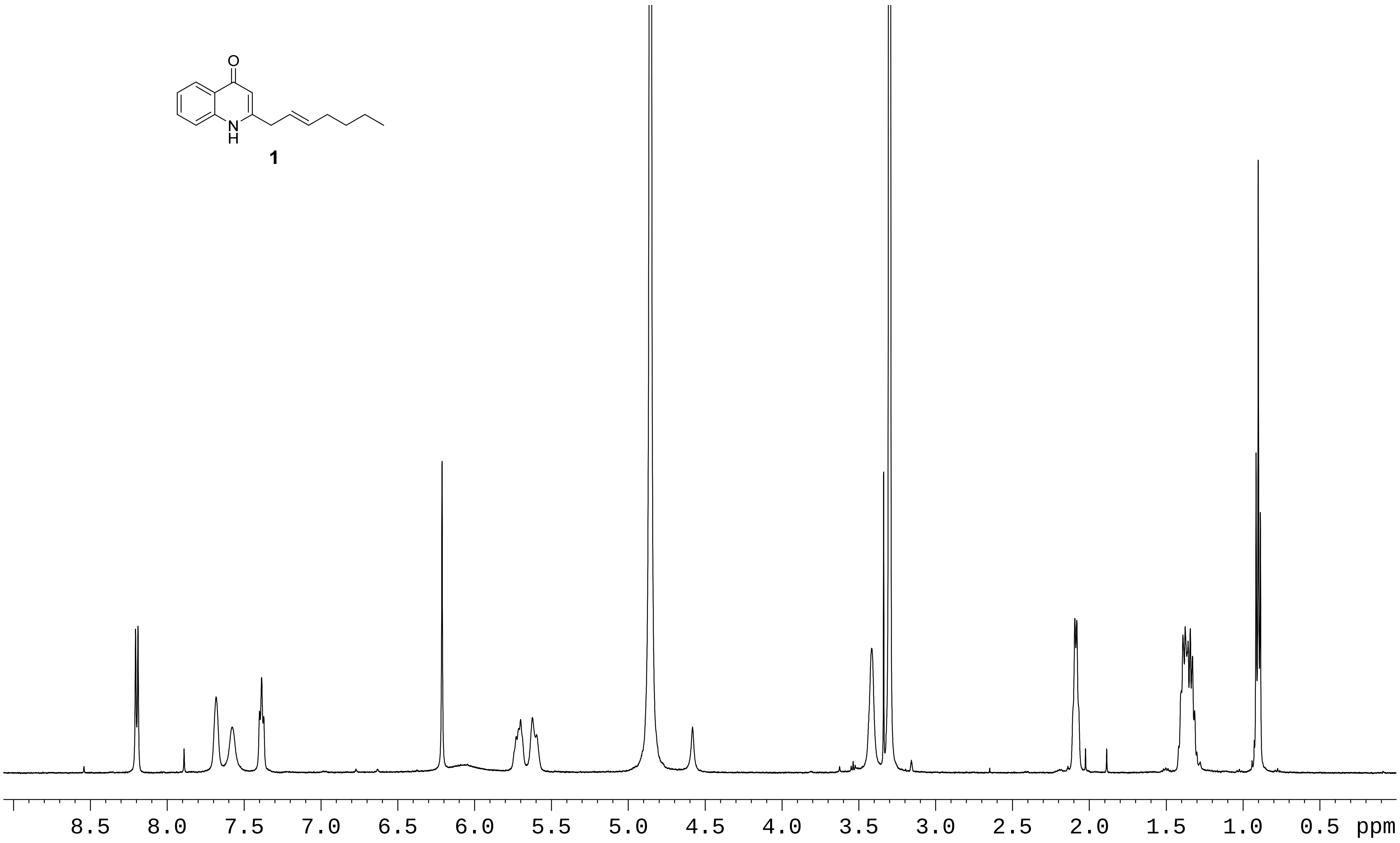

Figure S1: ^{1}H NMR spectrum of (*E*)-2-(hept-2-en-1-yl)quinolin-4(1*H*)-one (**1**, 500 MHz, CH_3OD)

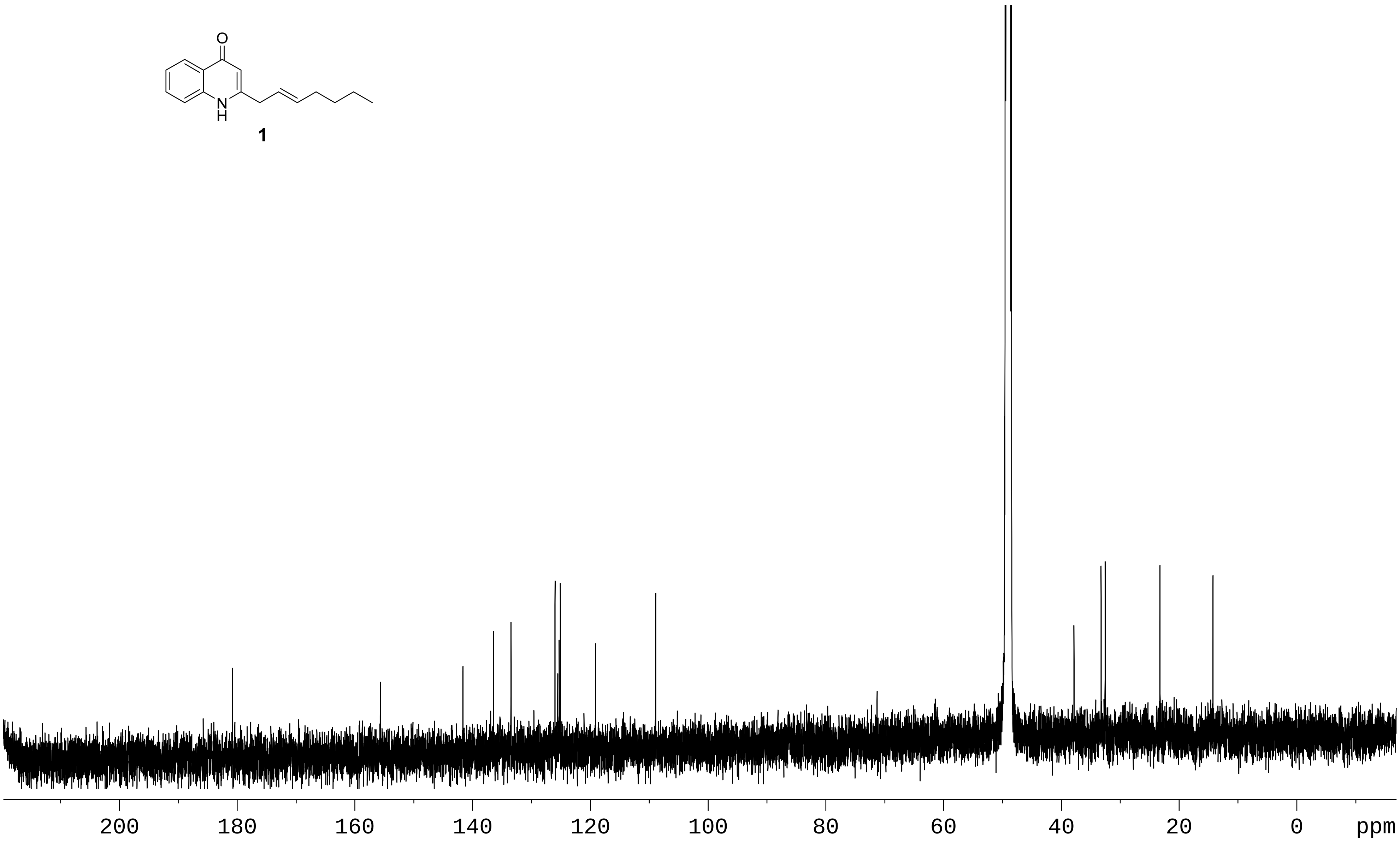

Figure S2: ^{13}C NMR spectrum of (*E*)-2-(hept-2-en-1-yl)quinolin-4(1*H*)-one (**1**, 125 MHz, CH_3OD)

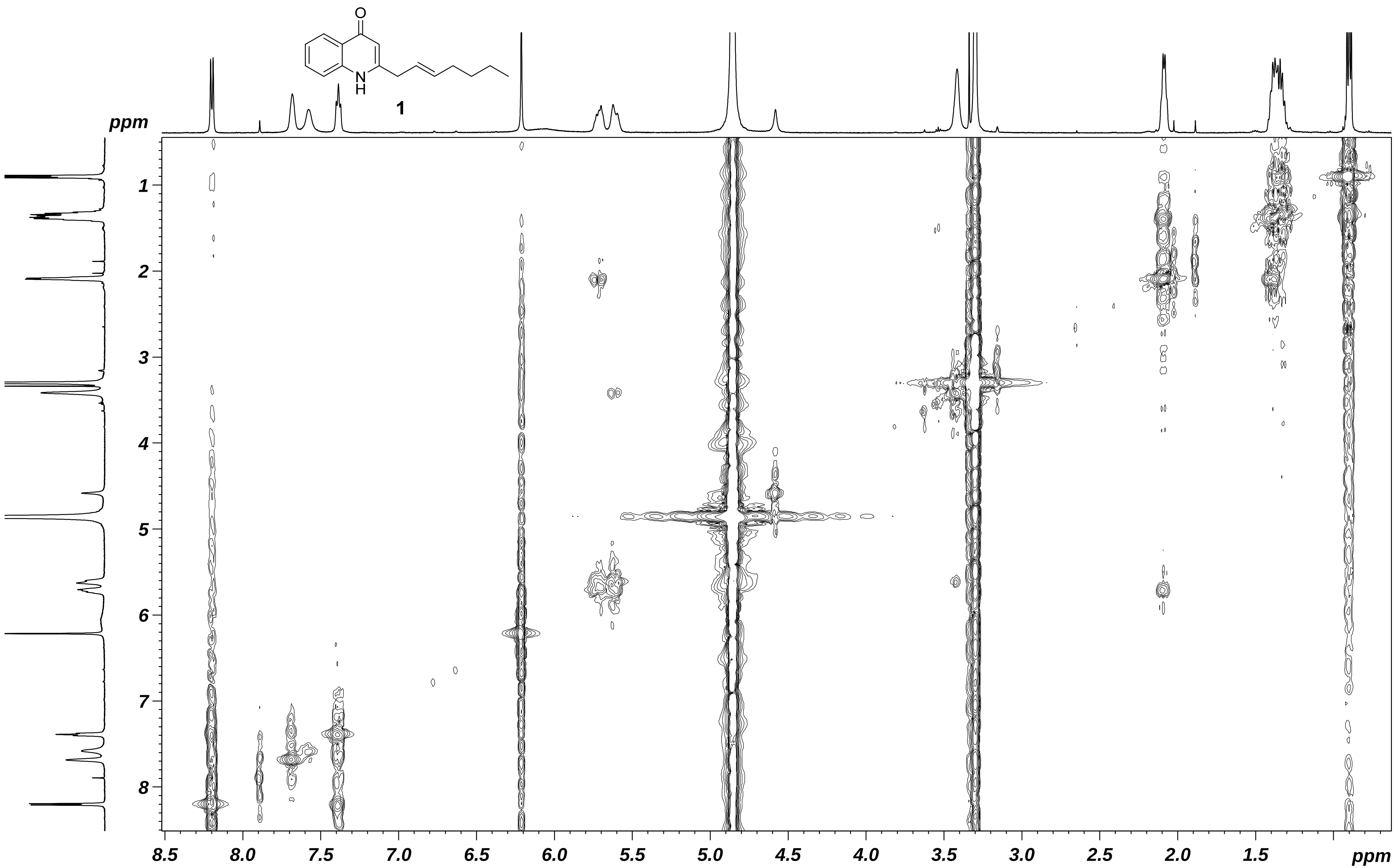

Figure S3: COSY spectrum of (*E*)-2-(hept-2-en-1-yl)quinolin-4(1*H*)-one (**1**, 500 MHz, CH_3OD)

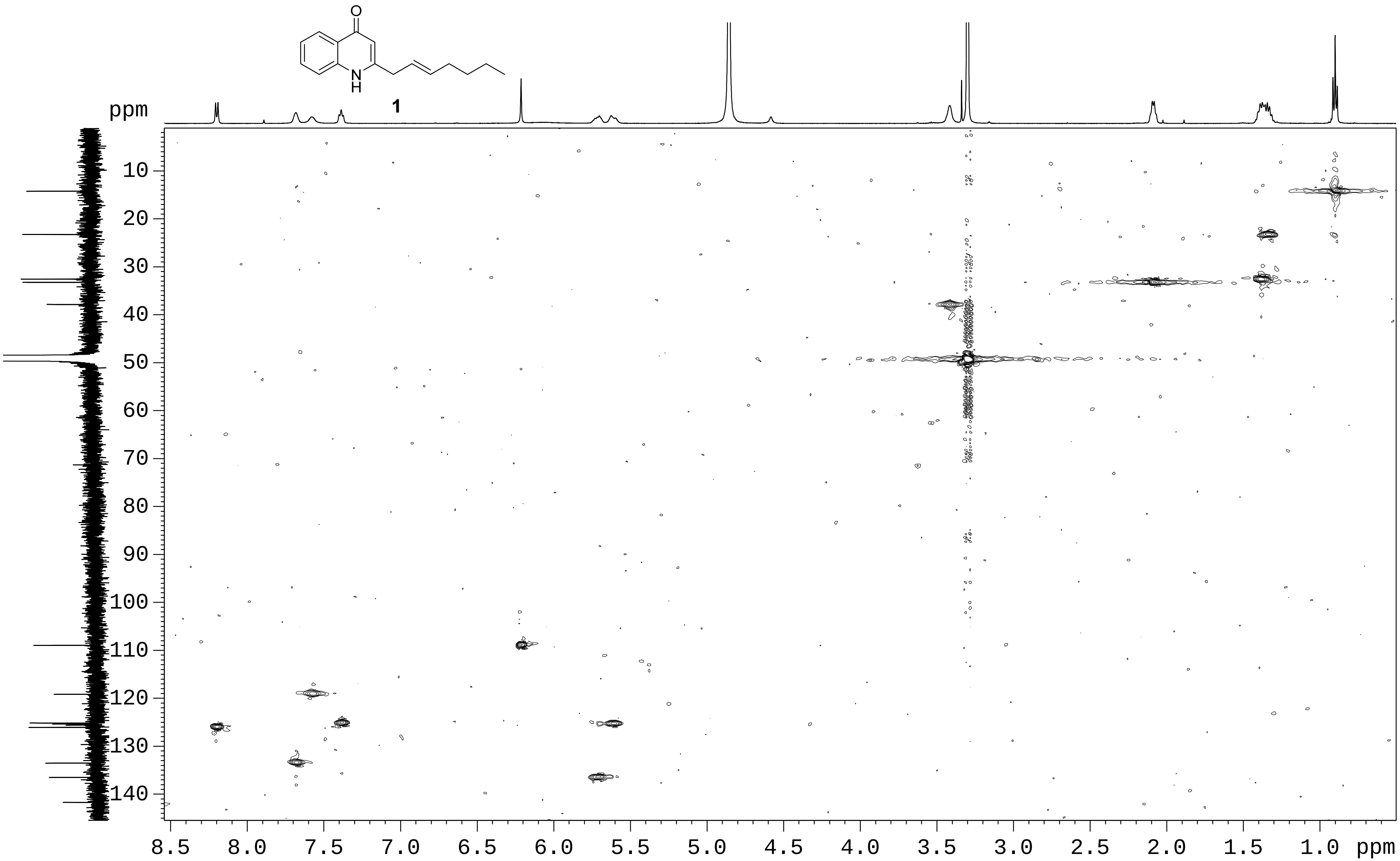

Figure S4: HSQC spectrum of (*E*)-2-(hept-2-en-1-yl)quinolin-4(1*H*)-one (**1**, 500 MHz, CH_3OD)

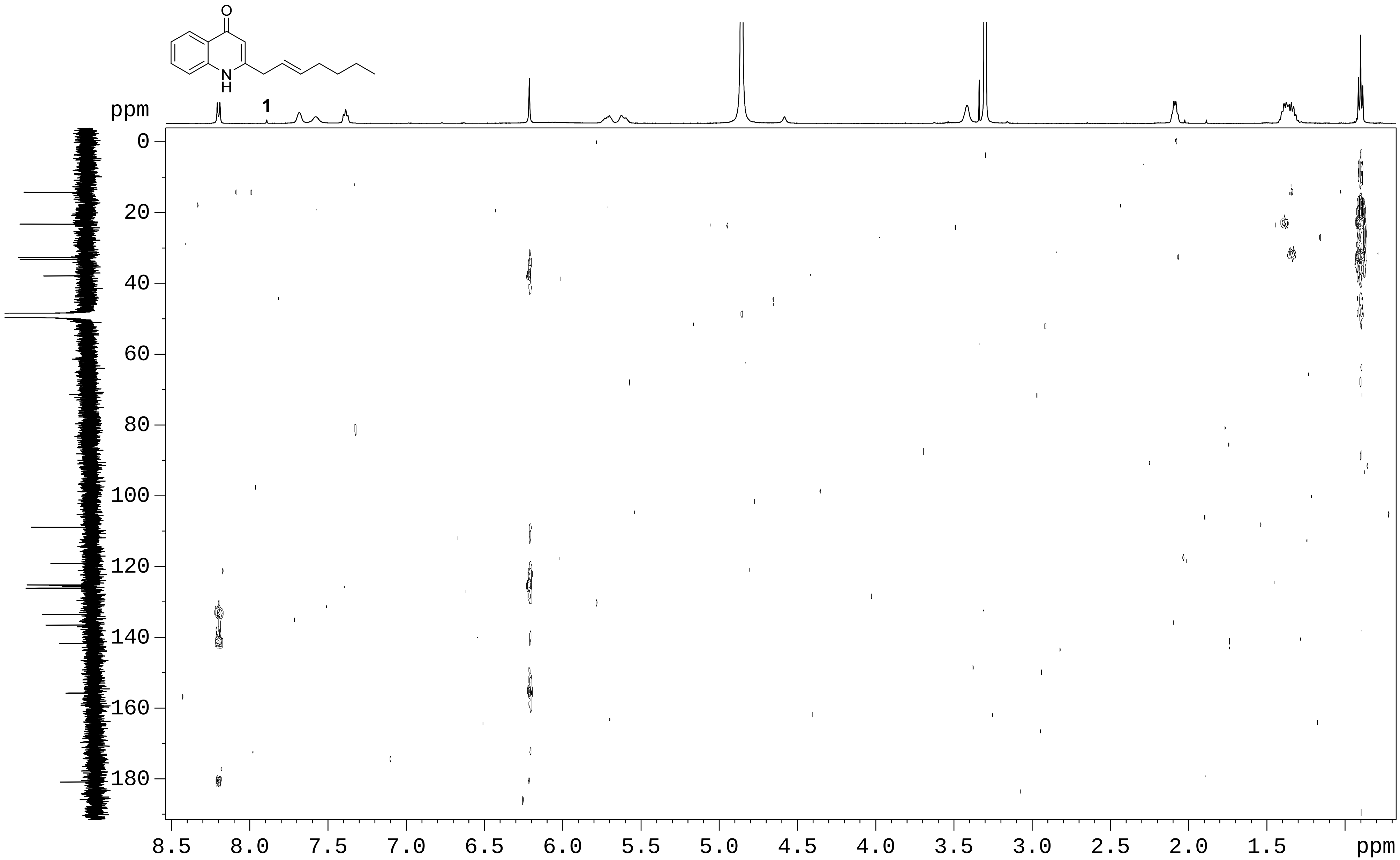

Figure S5: HMBC spectrum of (*E*)-2-(hept-2-en-1-yl)quinolin-4(1*H*)-one (**1**, 500 MHz, CH_3OD)

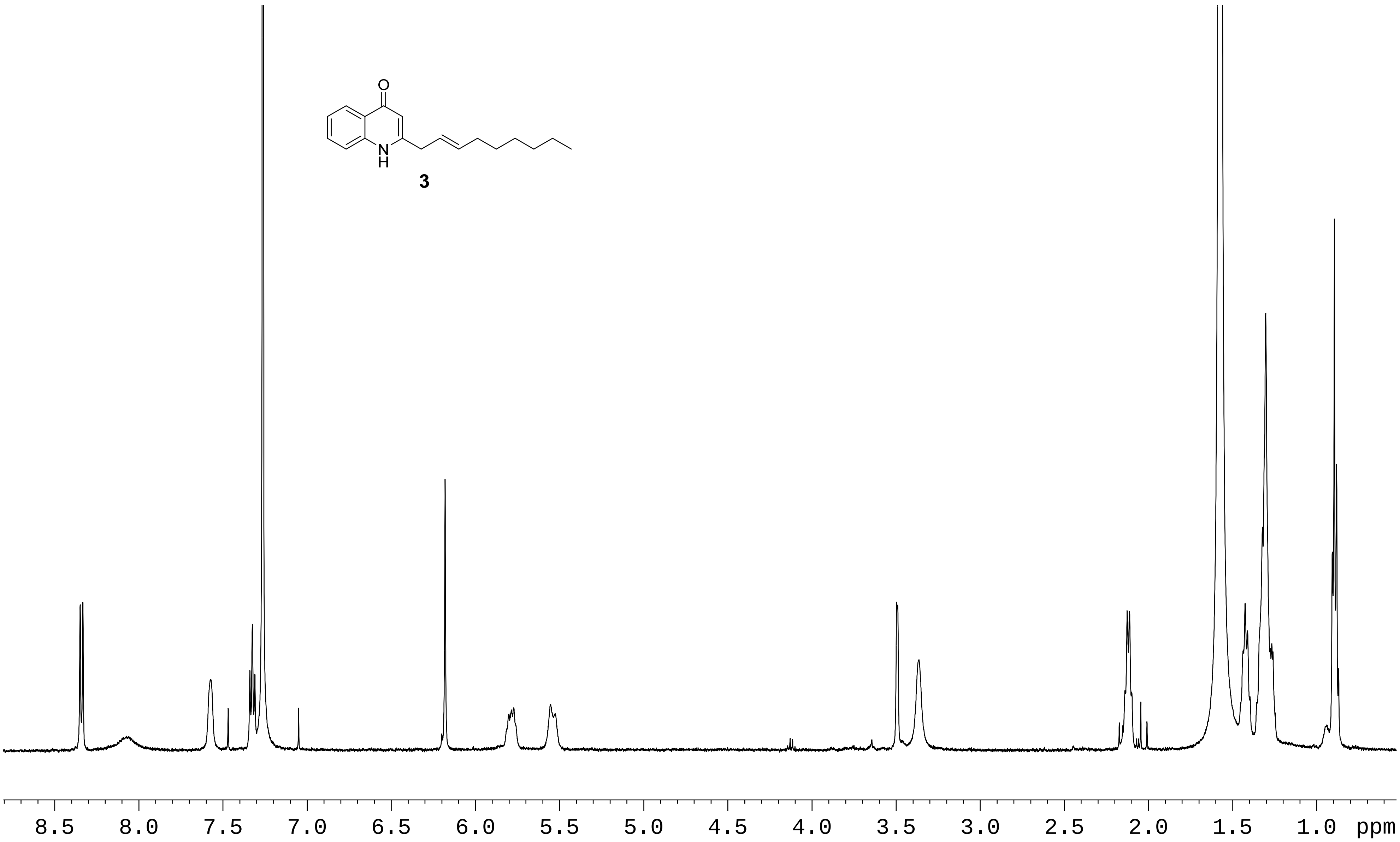

Figure S6: ^{1}H NMR spectrum of (*E*)-2-(non-2-en-1-yl)quinolin-4(1*H*)-one (**3**, 500 MHz, $CDCl_3$)

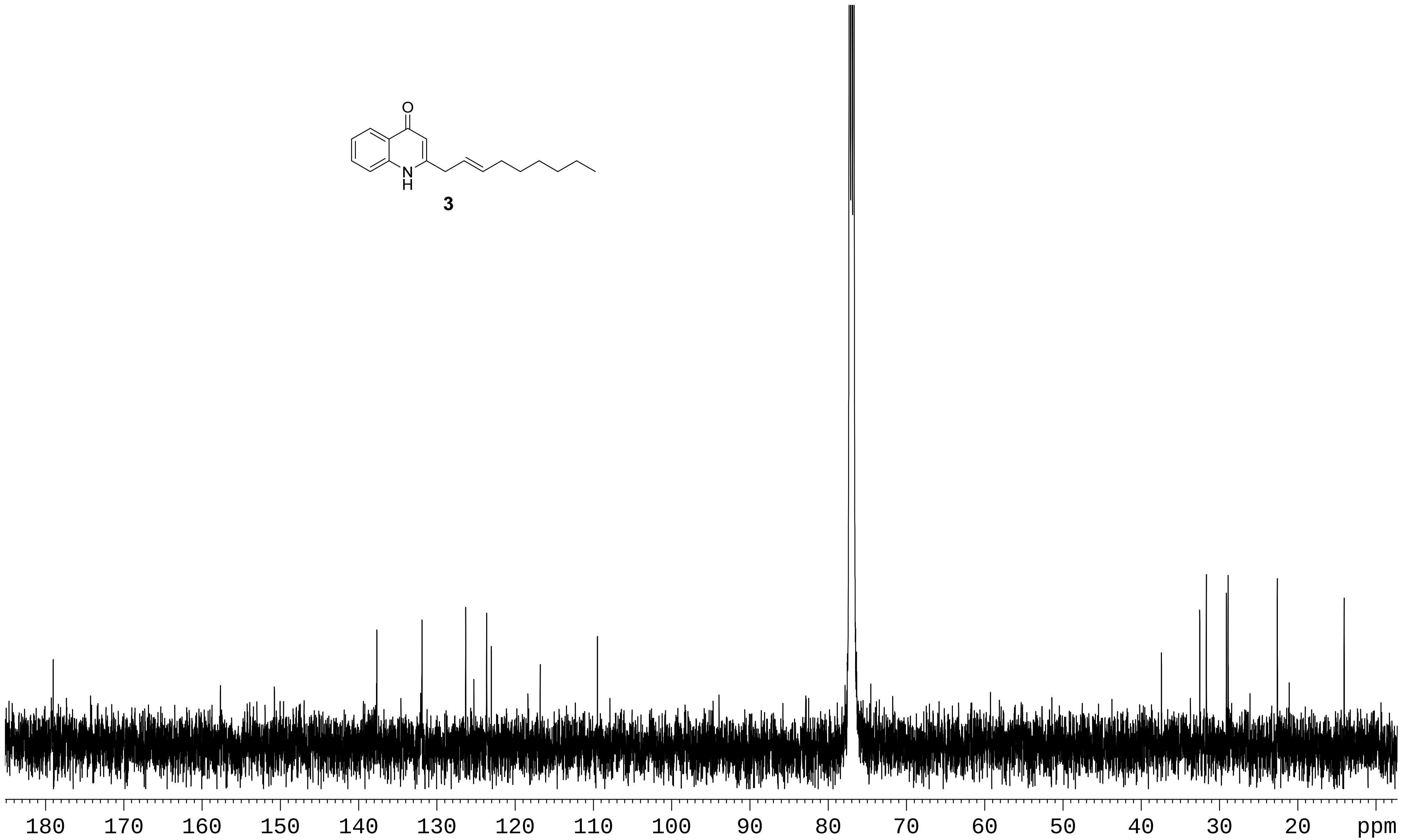

Figure S7: ^{13}C NMR spectrum of (*E*)-2-(non-2-en-1-yl)quinolin-4(1*H*)-one (**3**, 125 MHz, $CDCl_3$)

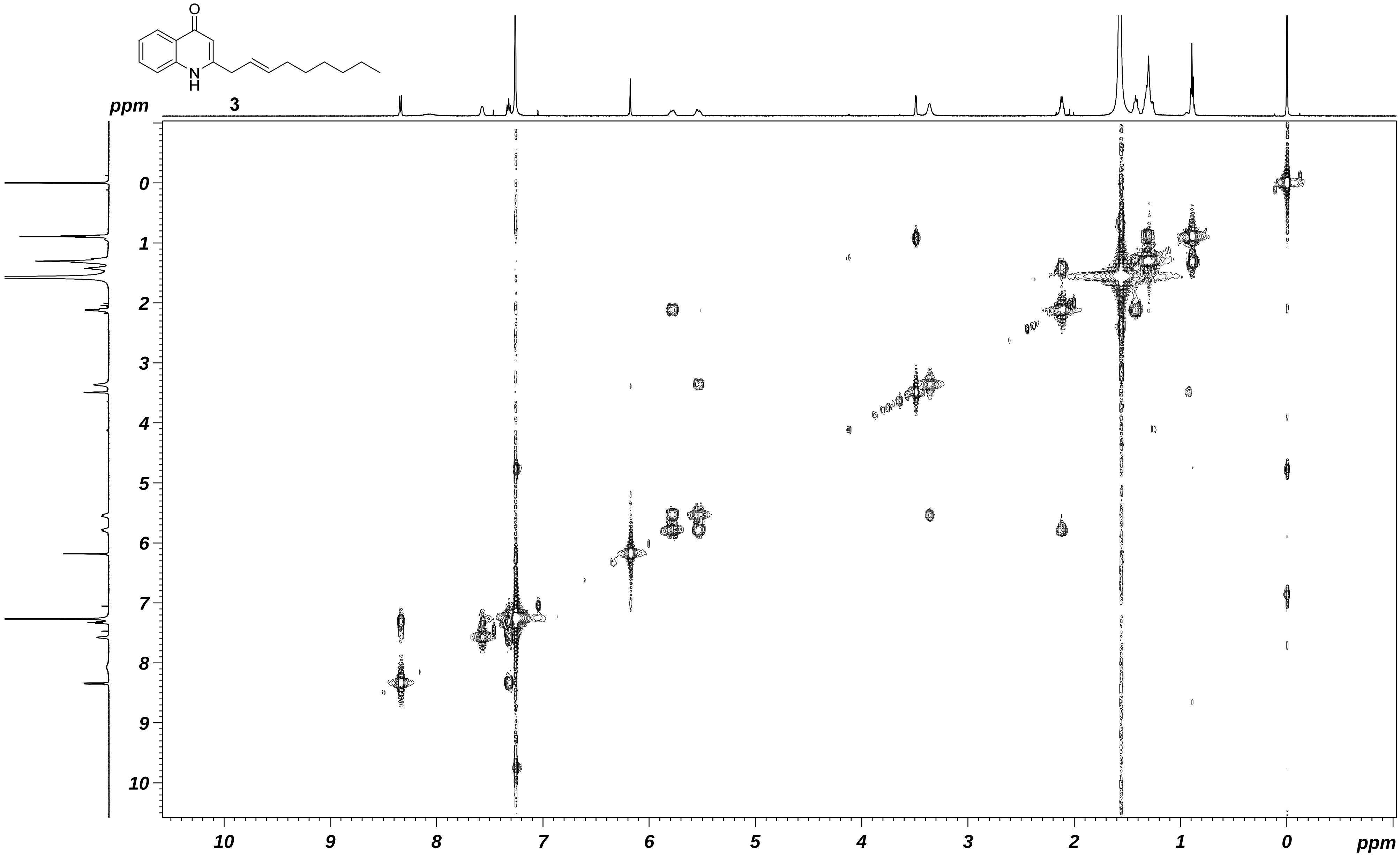

Figure S8: COSY spectrum of (*E*)-2-(non-2-en-1-yl)quinolin-4(1*H*)-one (**3**, 500 MHz, $CDCl_3$)

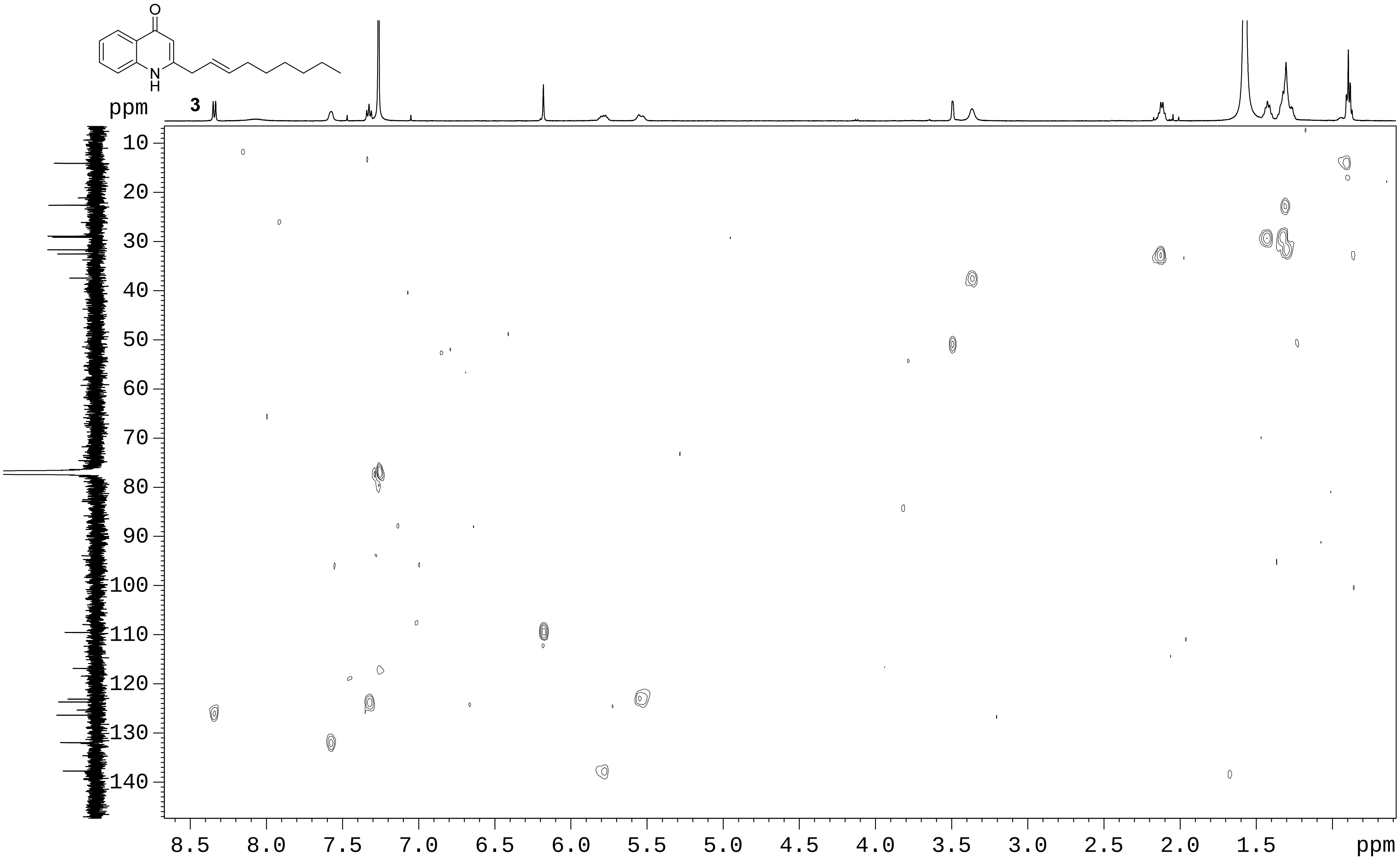

Figure S9: HSQC spectrum of (*E*)-2-(non-2-en-1-yl)quinolin-4(1*H*)-one (**3**, 500 MHz, $CDCl_3$)

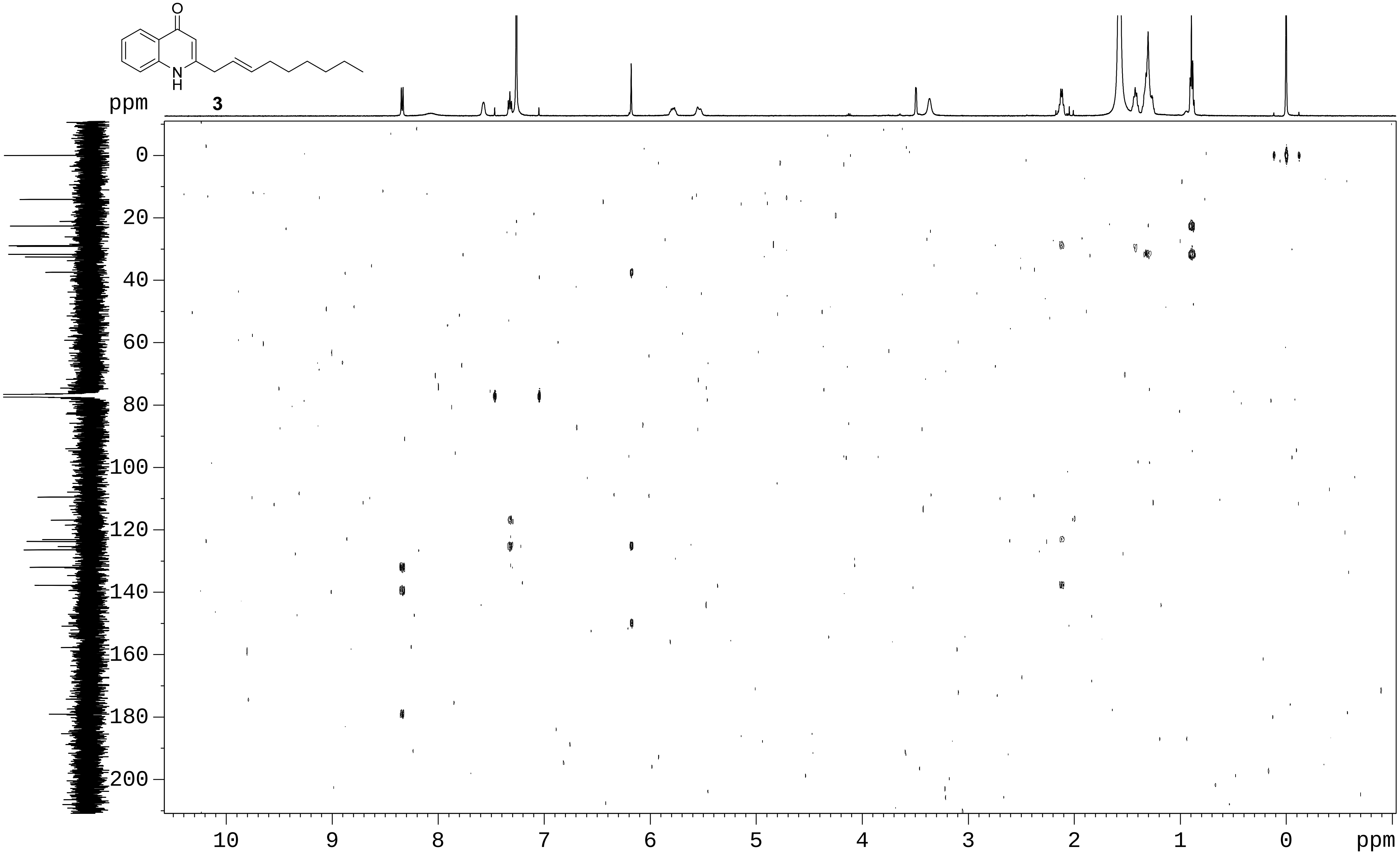

Figure S10: HMBC spectrum of (*E*)-2-(non-2-en-1-yl)quinolin-4(1*H*)-one (**3**, 500 MHz, $CDCl_3$)

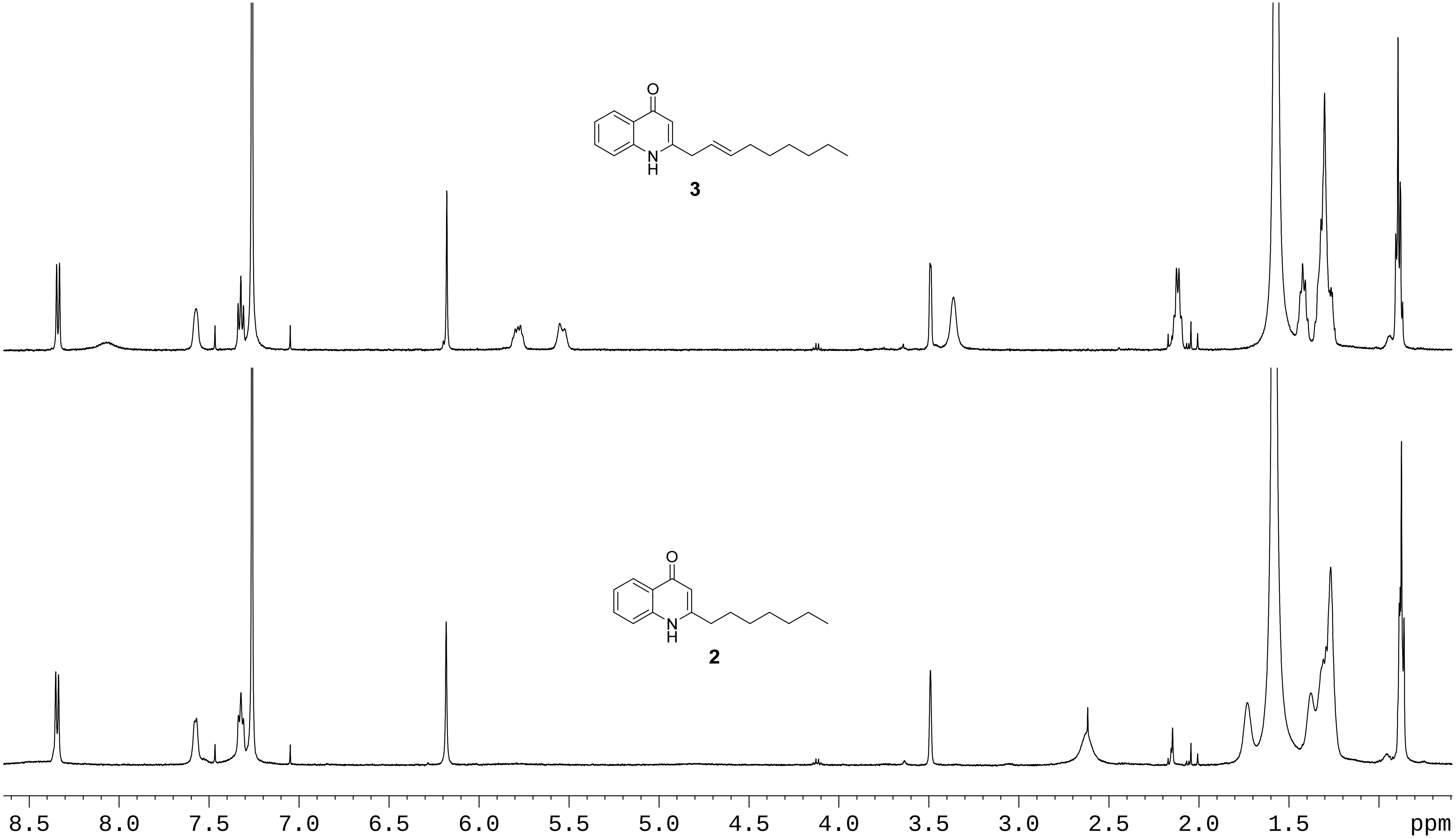

Figure S11: ^{1}H NMR spectral comparison between (*E*)-2-(non-2-en-1-yl)quinolin-4(1*H*)-one (**3**) and heptylquinolin-4(1*H*)-one (**2**, 500 MHz, $CDCl_3$)

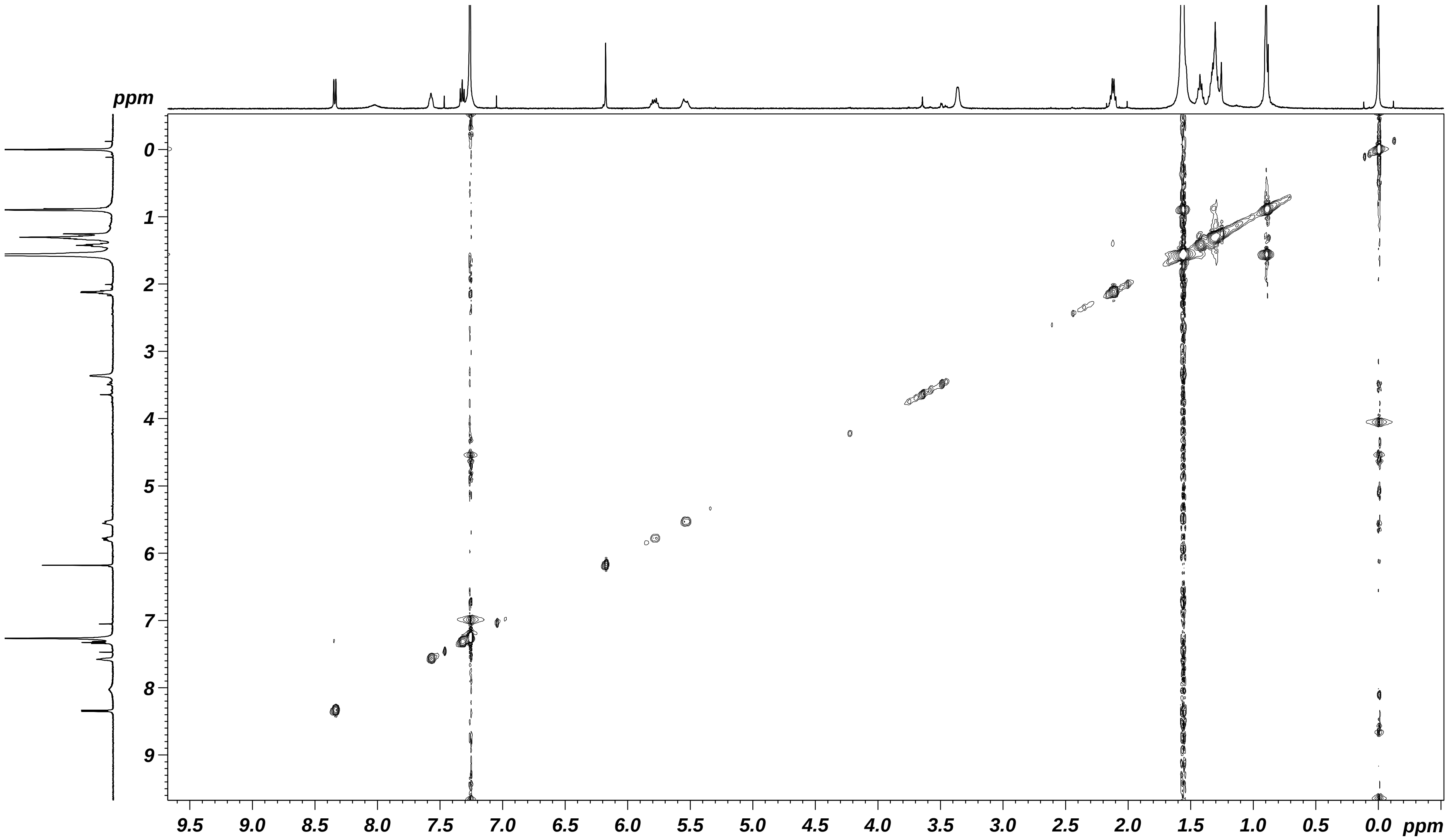

Figure S12: NOESY spectrum of (*E*)-2-(non-2-en-1-yl)quinolin-4(1*H*)-one (**3**, 500 MHz, $CDCl_3$, mixing time= 550 ms)
